# Bending and Straightening of a Medium Carbon Steel Continuous Casting Slab with Low Temperature End Plastic Groove

**DOI:** 10.3390/ma15072609

**Published:** 2022-04-01

**Authors:** Jingbo Yang, Fujun Zhang, Jingshe Li, Wei Liu, Tiantian Wang, Hang Yuan, Daqiang Cang

**Affiliations:** 1 School of Metallurgical and Ecological Engineering, University of Science and Technology Beijing, Beijing 100080, China; b20100188@xs.ustb.edu.cn (J.Y.); lijingshe@ustb.edu.cn (J.L.); wangtiantian@xs.ustb.edu.cn (T.W.); yuanhangneu@foxmail.com (H.Y.); cangdaqiang@metall.ustb.edu.cn (D.C.); 2 Beijing Key Laboratory of Special Melting and Preparation of High-End Metal Materials, Beijing 100080, China

**Keywords:** continuous casting, medium carbon microalloyed steel, corner transverse crack, third brittle interval

## Abstract

The high temperature brittleness range of medium carbon microalloyed steel under an actual continuous casting process was determined by the high temperature tensile test. The test results revealed that only a third of the brittle temperature range from 650–825 °C was due to intergranular ferrite in the experimental steel. In addition, it was found that the plastic recovery was fast and stable when the temperature was lower than 725 °C (the lowest plastic temperature). Bending/straightening operation in this temperature range was conducive to controlling the generation of corner cracks. In order to keep the corner temperature at the low temperature end of the plastic curve when the slab was bent/straightened, the cooling water scheme of the secondary cooling zone of the continuous caster was formulated by numerical calculation. By appropriately increasing the cooling water flow in the foot roll and the secondary cooling zones 1–5, the corner temperature of slab during bending operation was 600–700 °C, avoiding the brittle temperature range. The industrial test was then carried out. The results showed that after using the optimized water volume, the corner grains of the slab were uniform and the microstructure was mainly pearlite + ferrite. In addition, the abnormally large grain size was reduced, and a large amount of ferrite was generated inside the grain, which avoided stress concentration at the corner of the slab during bending/straightening operation, and basically eliminated the corner crack of the slab.

## 1. Introduction

The corner transverse crack of a continuous casting slab is universal, and a corner transverse crack with high incidence is generally a significant influencing factor of the surface quality of slabs [1,2]. In recent years, microalloying elements have been added to steel to improve its strength. Moreover, a straight arc continuous caster was used to promote the removal of inclusion. However, the straight arc continuous caster increased the bending operation, thus resulting in corner cracks of the outer arc side of the slab. Due to the complex formation mechanism of the corner transverse cracks and multiple influencing factors [3,4,5], its control technology has attracted significant research attention.

The high temperature brittleness of materials is essential because of corner cracks. The high temperature brittleness of materials has been widely studied, but most of them are aimed at low-carbon microalloyed steel [6,7,8]. At present, it is generally believed that there are three high temperature brittle temperature ranges within the continuous casting temperature. During continuous casting, the corner is cooled in two dimensions, and the corner temperature is generally in the third brittle temperature range of 700–900 °C due to the formation of ferrite thin film around the austenite grains [9,10]. The ductility of the high temperature end (higher than Ar_3_) decreases, and grain boundary slip plays a dominant role. At the same time, the precipitation free zone formed by fine carbonitrides between grains promotes grain boundary sliding, and pinning grains inhibits dynamic recrystallization and worsens the high temperature plasticity of the materials [11]. In low carbon steel containing more microalloying elements, the ductility reduction is dominated by precipitates, and the ferrite film has a less significant effect [12]. Therefore, changing the strain rate and the grain size reduces the precipitation of film-like ferrite and disperses the inclusions, which reduces the groove depth and improves the ductility of the entire temperature range [13]. However, due to the small variation range of strain rate during continuous casting and austenite grain size being greater than the critical value, and in order to improve the service life of the caster, most previous studies focused on controlling the precipitation process at the high temperature end of the groove and steel composition [11,14].

In the actual continuous casting production, the secondary cooling water flow is usually reduced, the temperature of the casting slab is increased, and the corner plasticity is improved [15,16]. In addition, the bending/straightening of the high temperature end reduces slab strength, thereby reducing caster load, wear, and bending stress. However, when the tensile temperature is higher than the lowest plastic temperature, the excellent thermoplastic behavior of the high temperature tensile specimen is deceptive. Under actual continuous casting conditions, the large grain size, small tensile strain, and low bending temperature inhibit the dynamic recrystallization behavior of the slab. The coarsening of precipitation or reduction in the precipitation amount may occur, thus resulting in slight ductility improvement [11]. Although a higher temperature can improve the plasticity of the slab, the plastic improvement is small and unstable, and corner cracks may still appear during bending/straightening. In the straight arc continuous caster, the slab undergoes significant cooling before bending, and the temperature is from 700–900 °C. Moreover, the groove temperature demonstrates excellent plasticity and requires extension to Ar_3_, which is close to 1000 °C. Such a high temperature is difficult to achieve during actual operation. The trough of the brittleness temperature cannot be avoided by increasing the slab corner temperature, and a lot of ferrite is formed in the low temperature end of the groove, which is 20–30 °C lower than the temperature of the Ar_3_ point. Moreover, the plasticity improvement is as high as 50% [17].

The high temperature brittleness range of medium carbon microalloyed steel under an actual continuous casting process was determined by the high temperature tensile test. Then, through numerical calculation, the cooling water scheme of the secondary cooling zone of the continuous caster was determined. The feasibility of the bending/straightening operation of the low temperature end of the continuous casting slab groove to eliminate corner cracks was explored through on-site industrial tests.

## 2. Materials and Methods

### 2.1. High Temperature Tensile Test

**Figure 1:**
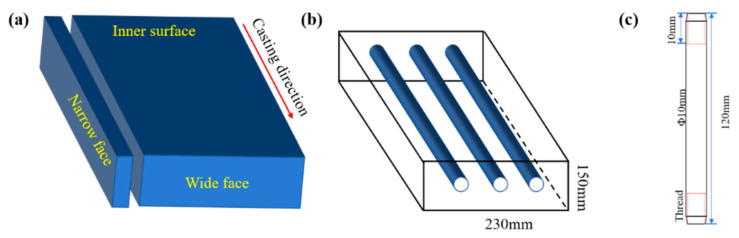
Schematic diagram of sampling and sample preparation. (**a**) Slab sampling location; (**b**) Sampling position of tensile specimen; (**c**) Schematic diagram of tensile specimen.

The samples used in the experiment were obtained from the slab at the production site. The composition of experimental steel is presented in Table 1. Figure 1a,b present schematic diagrams of the sampling. As illustrated in Figure 1c, the sample size for the hot tensile test was Φ × 10 mm × 120 mm.

**Figure 2:**
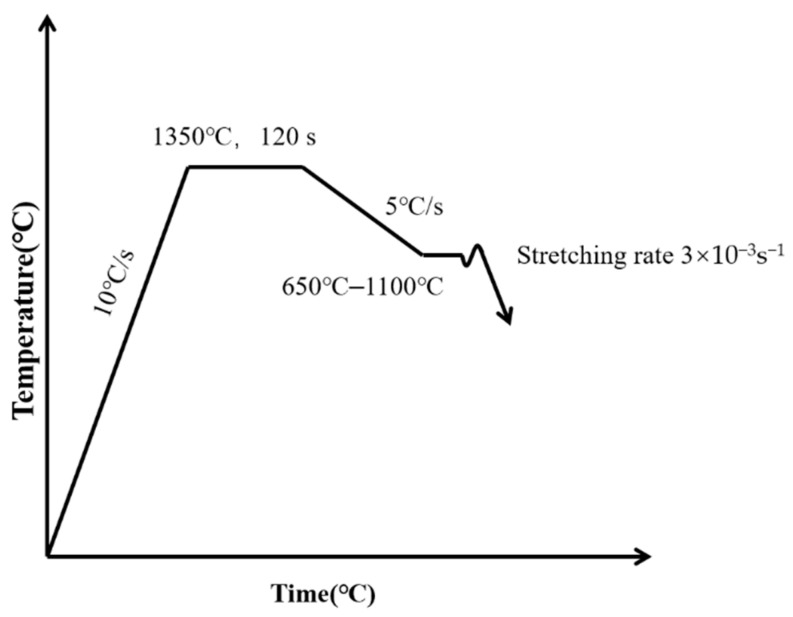
Hot tensile test scheme.

**Figure 3:**
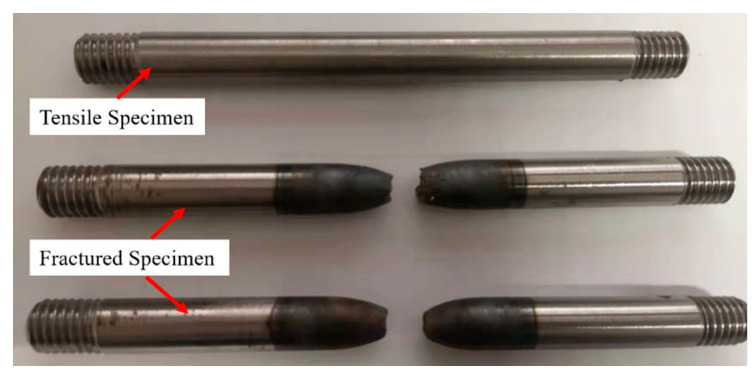
Picture of the sample during the tensile experiment.

According to the simulation results of the temperature field under the condition of the original secondary cooling water and the common casting speed, the temperature scheme and drawing rate of the high temperature tensile test are designed [18,19]. The temperature and tensile scheme of high temperature tensile test are shown in Table 2 and Figure 2. The specific experimental process was as follows: Firstly, the sample was installed in the predetermined position for argon filling protection to prevent high temperature oxidation. Then, the sample was heated to 1350 °C at a heating rate of 10 °C·s^−1^, kept for 160 s, completely austenitized, and then cooled to the tensile temperature at a cooling rate of 5 °C·s^−1^. After holding for 60 s, the sample was stretched at a strain rate of 3 × 10^−3^ s^−1^ until fracture. The fracture of the specimen during the tensile process is shown in Figure 3.

### 2.2. Numerical Simulation

#### 2.2.1. Assumption

1. Since the heat transfer in the casting direction of the mold accounts for only 3–6% of the total heat transfer, this part of heat transfer is ignored and only the heat transfer perpendicular to the casting direction is considered [20].

2. Converting convection heat transfer to heat conduction by increasing the thermal conductivity [20].

3. Ignoring the dimensional change of the slab during the solidification process of the continuous casting slab.

4. The heat transfer between mold flux and molten steel is not considered.

#### 2.2.2. Governing Equation

The two-dimensional transient thermal conductivity differential equation of solidification heat transfer is:

(1) $$\rho C_{p}\frac{\partial T_{\left(x,y\right)}}{\partial \tau }=\frac{\partial }{\partial x}\left(k\frac{\partial T_{\left(x,y\right)}}{\partial x}\right)+\frac{\partial }{\partial y}\left(k\frac{\partial T_{\left(x,y\right)}}{\partial y}\right)$$

where *T* is the temperature of the slab, in °C; *ρ* is the density of steel, in kg·m^−3^; *C_p_* is the specific heat capacity, in J·kg^−1^·K^−1^; *τ* is time, in s; *k* is the heat transfer coefficient, in W·m^−2^·K^−1^.

#### 2.2.3. Initial Condition

The molten steel pouring temperature at *τ* = 0 is defined as the molten steel temperature at the meniscus of the mold, that is, *T* = t_a_ (pouring temperature).

#### 2.2.4. Mold Boundary Conditions

The calculation of the heat flux density on the slab surface in the mold refers to the empirical formula (2) proposed by Savage and Pritchard [21]:

(2) $$q_{m}=A-B\sqrt{\frac{60z}{V_{c}}}$$

where *q_m_* is mold heat flux density, in W·m^−2^; *A* and *B* are constants; *z* is the distance to the meniscus, in m; *V* is the casting speed, in m·min^−1^.

Due to the corner being cooled in two dimensions, the coefficient m is introduced to correct the corner heat flux, as shown in Equation (3):

(3) $$q_{c}=mq_{m}$$

where *q_c_* is corner heat flux density, in W·m^−2^; *m* is the correction coefficient, with a value range of 0–1. 0–0.1 H below the meniscus (H is the effective length of the mold) is the tight zone, m is 1, 0.1–0.25 H below the meniscus is the air gap formation zone, *m* is 0.8; the rest is the stable zone, *m* is taken as 0.6.

#### 2.2.5. Boundary Conditions of the Second Cooling Zone

##### Pinch Roll Heat Transfer

The contact heat transfer between the pinch roller and slab surface is related to the contact area between roller and slab surface, slab surface temperature, and casting speed. The heat flux between roller and slab surface used in this simulation is shown in Equation (4) [19]:

(4) $$q_{r}=11513.7\times T_{s}^{-0.76}\times {\left(\frac{V_{c}}{60}\right)}^{-0.2}\times a^{-0.17}$$

where *q_r_* is the heat flux density of the pinch roll, in W·m^−2^; *T_s_* is temperature of the slab, in K; *a* is the angle corresponding to the arc length of the contact part between the pinch roll and the slab surface in °.

##### Radiation Heat Transfer

The relationship between radiant heat transfer per unit area and surface temperature follows the Stefan–Boltzmann law, as shown in Equation (5) [22]:

(5) $$q_{rad}=\delta \varepsilon {\left(T+273\right)}^{4}$$

where *q_rad_* is the heat flux density of radiation heat transfer, in W·m^−2^; *σ* is the Stefan–Boltzmann constant, and its value is 5.6684 × 10^−8^ W·m^−2^·K^−4^; *ξ* is the radiation coefficient, which is between 0 and 1.

##### Spray Water Heat Transfer

The heat flux density between the spray water and the surface of the continuous casting slab is shown in Equation (6) [23]:

(6) $$q_{w}=h\left(T_{s}-T_{w}\right)$$

where *q_w_* is the heat flux density of cooling water heat transfer, in W·m^−2^; *T_w_* is the cooling water temperature, in K; *h* is the heat transfer coefficient, in W·m^−2^·K^−1^.

#### 2.2.6. Thermophysical Parameters

The main parameters involved in the simulation calculation include density, solid–liquid phase line temperature, thermal conductivity, and specific heat capacity. The specific calculation equation is shown in Table 3.

## 3. Results and Discussion

### 3.1. Steel Grade Brittle Temperature Range

**Figure 4:**
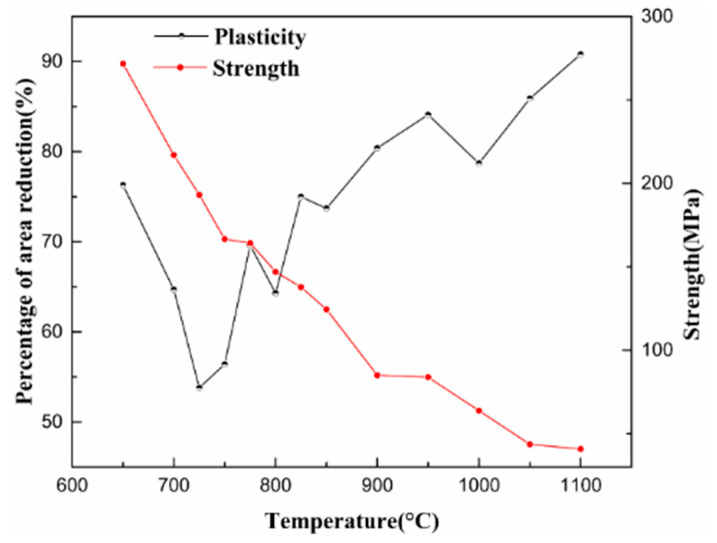
Thermoplasticity and strength curve of specimen.

**Figure 5:**
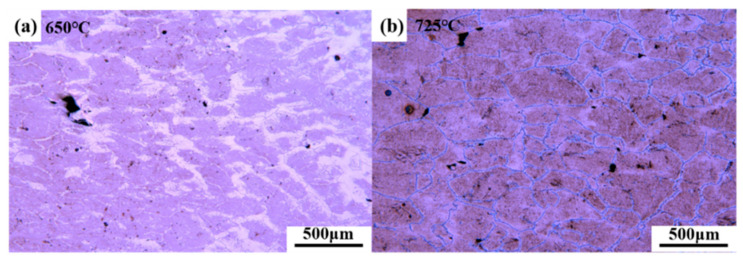
Microstructure of tensile specimen. (**a**) Microstructure of 650 °C tensile specimen; (**b**) Microstructure of 650 °C tensile specimen.

In the temperature range of 650–1100 °C, the strength decreased with an increase in temperature. As illustrated in Figure 4, at 900 °C and 775 °C, dynamic recrystallization [9] and intergranular ferrite formation [8] occurred, respectively. Two inflection points were generated to divide the strength curves. In the three temperature ranges of 650–750 °C, 750–900 °C, and 900–1100 °C, the strength increased by 10.51 MPa, 8.16 MPa, and 4.42 MPa, respectively, for each increment of 10 °C. The experimental steel contained a small amount of Ti, the control of Al and N elements was low, and the amount of carbonitride precipitation was significantly low. There was no second brittle temperature range caused by the precipitation of carbonitride in the steel. The third brittle interval (650–825 °C) was caused by the eutectoid ferrite. As shown in Figure 5, a large amount of ferrite appeared in the crystal at 650 °C at the low temperature end, and the plasticity was restored rapidly. In addition, the plastic instability at the high temperature end depended on the ferrite thickness between grain boundaries. At 725 °C, the original austenite grain boundary was covered with film ferrite, formed a network structure, and RA reached a minimum value of 50.1%.

### 3.2. Surface Temperature Distribution of Casting Slab

**Figure 6:**
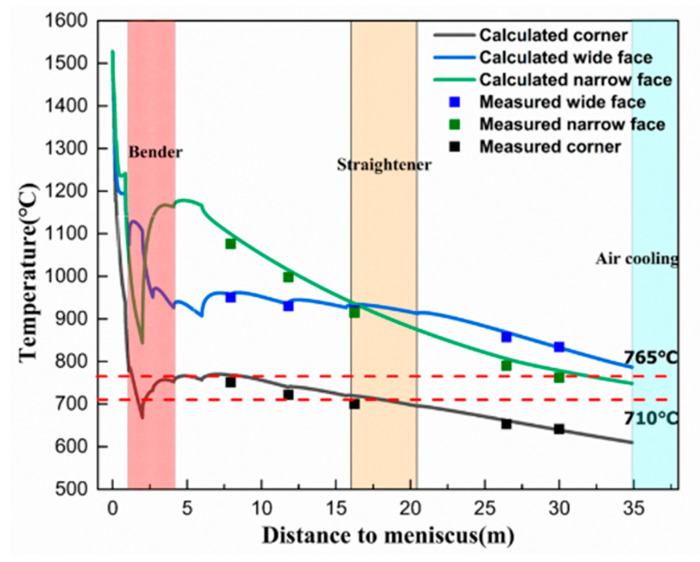
Surface temperature field of continuous casting slab.

Based on the fundamental theory of solidification heat transfer, the transient thermal module of ANSYS was used to establish a slab solidification heat transfer model. The surface temperature distribution of the slab was calculated, and a FLUKE handheld thermal imager (ranging from −20 °C to 1000 °C, with an accuracy of 2%) was used to measure the multi point temperature of the continuous casting slab on site to verify the accuracy of the calculation model. As shown in Figure 6, the calculated results of the temperature distribution of slab under the original water flow process was in good agreement with the measurement results, and the maximum error was within ±2%. The simulation calculation results reflected the temperature distribution of the slab under different secondary cooling water distribution schemes.

The rate of the cooling zone in the mold was relatively large, and the surface temperature of the continuous casting slab exhibited a rapid downward trend from an initial value of approximately 20 °C·s^−1^ to 0 °C·s^−1^ (the mold outlet), and the average cooling rate was 7 °C·s^−1^. In the secondary cooling zone, the cooling rate of the foot roll section was high (3–6 °C·s^−1^), and the cooling rate of the other zones decreased gradually. At a distance of approximately 2 m from the meniscus, the corner temperature, wide surface center temperature, and narrow surface center temperature of the continuous casting slab decreased from 937 °C, 1191 °C, and 1240 °C at the mold outlet to 667 °C, 1106 °C, and 843 °C, respectively. The corner temperature suited the temperature conditions of intergranular ferrite precipitation, and the centers of wide and narrow surfaces were in the single phase region of austenite. In addition, due to the reduction in cooling water, the temperature began to return. The corner was cooled by two-dimensional cooling and the temperature return speed was low. Moreover, the narrow surface water cooling was terminated after the foot roll was removed; the temperature return in the center of the narrow surface was large. The continuous casting slab was bent at the beginning of 2.33 m, and the temperatures of the corner, wide surface center, and narrow surface center were 720 °C, 996 °C, and 1087 °C, respectively. When leaving the bending section, the temperature recovery of the corner, wide surface center, and narrow surface center was completed; and the temperatures were 758 °C, 937 °C, and 1171 °C, respectively. When entering the straightening zone, the temperature decrease rate at the corners was 0.1–0.3 °C·s^−1^. Moreover, the temperatures of the corners, the center of the wide face, and the center of the narrow face were 719 °C, 933 °C, and 939 °C, respectively. The temperature when exiting the straightening zone were 695 °C, 912 °C, and 874 °C, respectively. Under the raw water process conditions, the surface center temperature of the bending/straightening section ranged from 850–1150 °C. Although the strength of the continuous casting slab was low, the thermoplasticity was excellent, and there were no surface cracks. When the corner entered the bending/straightening section, the corner temperature was in the brittle temperature range of 710–765 °C (RA was less than 60%), and the corner temperature was maintained at a high temperature for a long time, which had not reached the precipitation temperature of the intragranular ferrite. On the contrary, it promoted the growth of intergranular ferrite iron, formed a ferrite film, and the plasticity decreased sharply.

### 3.3. Microstructure of Continuous Casting Slab

**Figure 7:**
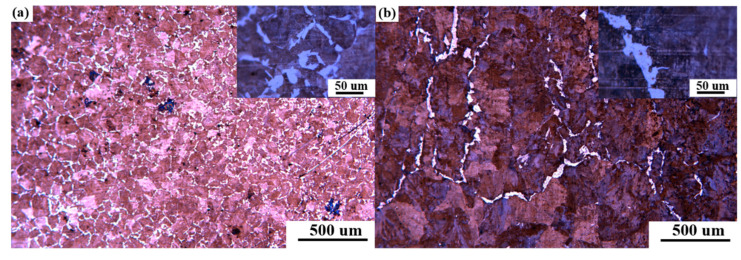
Microstructure of the corner of the continuous casting slab. (**a**) Microstructure of the corner of the ‘oscillation mark’ area; (**b**) microstructure of the corner of the ‘non-oscillation mark’ area.

The original austenite grain size was dependent on the cooling intensity in the mold [27,28], and the mold vibration formed a periodic ‘oscillation mark’ on the surface of the continuous casting shell [29], thus deteriorating the shell heat transfer. At the same time, due to the ‘oscillation mark’ in the secondary cooling zone, intermittent contact between the secondary cooling rolls and the surface of the slab, and the uneven cooling of the nozzles, abnormally coarse austenite grains were produced on the surface of the slab. As illustrated in Figure 7 (microstructure of the corner of the slab), under the original secondary cooling water distribution scheme, the austenite grain size on the corner surface of the slab was not uniform. In the excellent heat transfer area, the local grain size was uniform (approximately 100 μm), and ferrite was precipitated at the grain boundary (Figure 7a). Figure 7b reveals that the crystal grains at the ‘oscillation mark’ were as large as 500 μm, and several were larger than 1 mm. At the same time, 25–50 μm film-like ferrite particles of different thicknesses were precipitated at the grain boundaries, which deteriorated the high temperature ductility of the continuous casting slab corners.

### 3.4. Optimization of the Secondary Cooling Water Distribution Scheme

The cooling scheme of the secondary cooling zone follows the principle of target temperature control. In addition, the cooling rate in the length direction of the slab should not exceed 200 °C·m^−1^ and the temperature increase rate should not exceed 100 °C·m^−1^ for the prevention of cracks caused by excessive thermal stress in the slab [26]. The target temperature was obtained using the Gleeble 3500 thermal simulator and the temperature of the second cold zone was controlled by matching the target temperature based on a simulation calculation of the temperature field to obtain the corresponding cooling water flow.

As shown in Table 4, by changing the cooling water flow rate in the foot roll section and the secondary cooling sections 1–5, the corner temperature of the slab entering the bending/straightening section could avoid the 710–765 °C brittleness interval.

**Figure 8:**
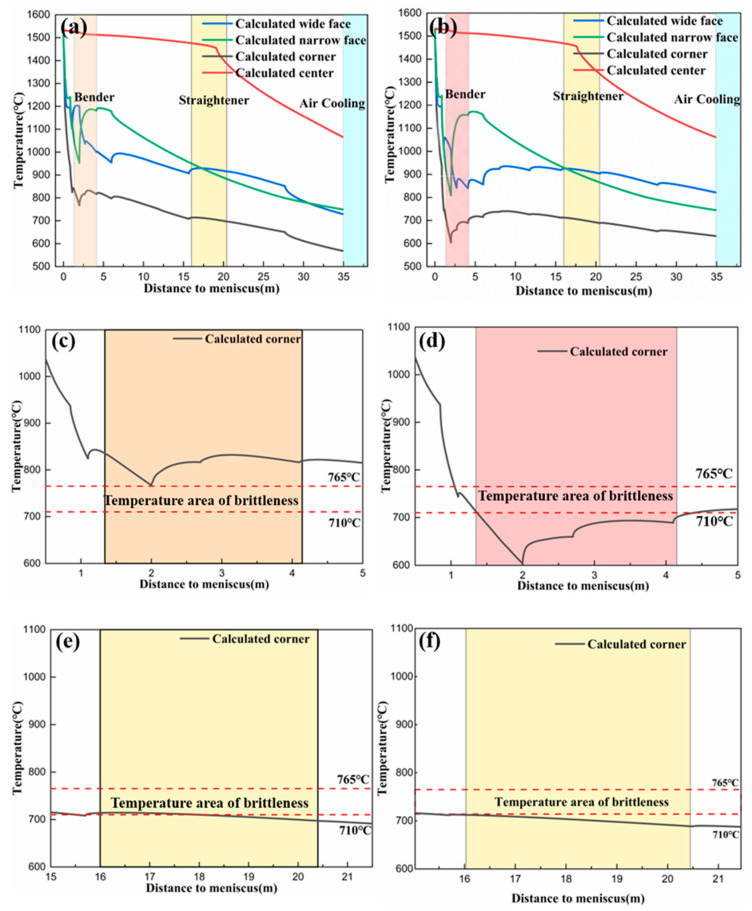
Continuous casting slab temperature curve under different water content conditions. (**a**,**c**,**e**) represent the temperature curves of the whole, bending section, and straightening section under weak cold conditions; and (**b**,**d**,**f**) represent the temperature of the whole, bending section, and straightening section under strong cold conditions.

The bending/straightening operation at the high temperature end was adopted. As shown in Figure 8a,c,e, under the condition of weak cooling, the temperature changes of casting slab as a whole, bending section, and straightening section were respectively shown. The temperature at a distance of 2 mm from the meniscus decreased to its lowest value and the surface temperature of the casting slab entering the bending section was in the process of temperature recovery. When the casting slab was bent at a temperature higher than 765 °C, intragranular ferrite was not formed, and intergranular ferrite grew rapidly. Moreover, the water volume was reduced significantly, and the specific water volume was only 0.56 L·kg^−1^. With the exception of the lowest temperature point, the temperature was generally higher than 800 °C. The surface center temperature of the continuous casting slab in the bending/straightening section was 900–1200 °C, which was prone to bulging, center segregation, and other quality defects. The corner temperature was at 765–825 °C at the high temperature end, the reduction of area fluctuated around 65%, and the increase in plasticity was low and unstable. In addition, in the straightening section, the corner was at the lower limit of the brittle temperature range.

The bending/straightening operation at the low temperature end was adopted. As illustrated in Figure 8b,d,f, under the condition of strong cooling, the temperature changes of casting slab as a whole, and bending and straightening sections were shown, respectively. By increasing the water flow of the secondary cooling zone and adjusting it properly, the temperature change was stabilized. Compared with the weak cooling, the surface center temperature during bending was 800–1150 °C, which was reduced by 50–100 °C. The corner temperature was 600–700 °C, which was reduced by 100–150 °C. The temperature of slab straightening was lower than the brittle temperature range. The bending and straightening operations were performed at the low temperature end. The corners of the cast slab were in the low temperature zone throughout, and the lowest temperature reached 600 °C. The intergranular and intragranular ferrite grew uniformly, the plasticity was improved and stable, and the brittle temperature range was effectively avoided. Moreover, the central temperature was reduced by 100–150 °C. To ensure the strength of the slab shell, the load of the continuous casting machine could not be increased significantly.

### 3.5. Application Result

#### 3.5.1. Test Results

By considering the plastic temperature range obtained from the Gleeble 3500 thermal simulation test results as the target temperature, combined with the continuous casting slab temperature field simulation calculation, a strong cooling scheme was used to optimize the secondary cooling water flow of the straight arc continuous casting, such that the slab entered the bending/straightening section when the surface center temperature was controlled from 800–1150 °C. Moreover, the corner surface temperature was 600–700 °C.

Industrial comparative tests were carried out on a two-strand, straight arc slab caster with different secondary cooling water distribution schemes. However, because the specific water volume of the weak cooling water scheme was too small, the strong cooling scheme was selected to optimize the water volume in order to prevent pouring accidents. The experimental results were shown in Table 4.

The test results are presented in Table 5. There were 12 slabs in Test 1, with no observable corner transverse cracks. In the non-experimental strand, 40 slabs within Slabs 2 had corner transverse cracks, and the defect rate was 5%. Corner transverse cracks occurred in 10 of the 52 slabs in the two strands with a defect rate of 19%, thus indicating that the formation of the corner transverse cracks could be effectively controlled by appropriately increasing the cooling water before the bending section, the thickness of the grain boundary ferrite film, and the formation of intragranular ferrite.

#### 3.5.2. Microstructure of the Corner of the Continuous Casting Slab

**Figure 9:**
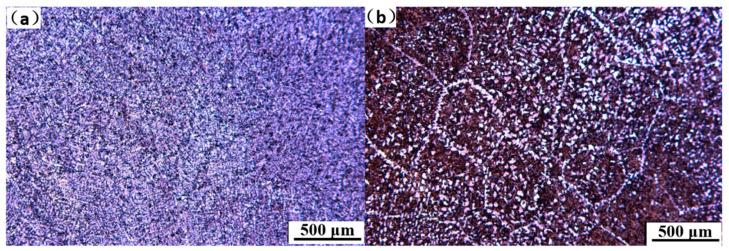
Microstructure of the corner of the continuous slab. (**a**) Microstructure of the corner of the ‘oscillation mark’ area, and (**b**) microstructure of the corner of the ‘non-oscillation mark’ area.

After the bending/straightening process was carried out at the low temperature end, as illustrated in Figure 9a, the overall grain size of the continuous casting slab was uniform and mainly composed of ferrite and pearlite. As illustrated in Figure 9b, in several abnormal grain areas, the precipitation of ferrite in the crystal was increased, the corner plasticity was improved, and the incidence of corner transverse cracks was reduced.

## 4. Conclusions

(1) In the temperature range of 650–1100 °C, strength decreased with an increase in temperature, and there was a brittle temperature range of 650–825 °C. At 900 °C, the dynamic recrystallization strength decreased and the plasticity increased. At 775 °C, ferrite was formed between the grains, and strength and plasticity were significantly reduced.

(2) The experimental steel contained a small amount of Ti, and the control of Al and N elements was low. Moreover, the amount of carbonitride precipitation was significantly low, and there was no second brittle temperature range caused by the precipitation of carbonitride in the steel. Under the raw secondary cooling water conditions, when the continuous casting billet entered the bending/straightening section, a network-like ferrite film was formed on the coarse grains, and plasticity was significantly reduced.

(3) The bending/straightening process at the low temperature end was adopted. By increasing the water volume of the foot roll section and the secondary cooling zones 1–5; In the bending/straightening sections, the slab surface center was controlled at 800–1150 °C, the corner surface temperature was 600–700 °C, and the overall grain size of the continuous casting slab was uniform. It was mainly composed of ferrite and pearlite. In several abnormal grain areas, the amount of ferrite precipitates in the crystal increased. To ensure the strength of the slab shell and the load of the continuous casting machine, the corner plasticity was improved, and the incidence of corner transverse cracks was reduced.

## Figures and Tables

**Table 1:** Composition of experimental steel (mass fraction, %).

C	Si	Mn	P	S	Al	Ti	N	Cr	Ni	Mo	Ca
0.2945	0.1988	0.5122	0.0181	0.0019	0.0221	0.015	0.00417	0.97	0.1292	0.1673	0.0019

**Table 2:** Experimental Temperature Scheme.

Test Temperature (°C)												
650	700	725	750	775	800	825	650	700	725	750	775	800

**Table 3:** Main thermophysical parameters.

Parameter	Equation	Method
Solidus	$\begin{array}{cc} T_{L}=1537- & \{88\left(\%C\right)+25\left(\%S\right)+5\left(\%Cu\right)+8\left(\%Si\right)+5\left(\%Mn\right)\\ & \;+2\left(\%Mo\right)+4\left(\%Ni\right)+1.5\left(Cr\right)+18\left(\%Ti\right)+2\left(\%V\right)\\ & \;+30\left(\%P\right)\} \end{array}$	Empirical formula [24]
Liquidus	$\begin{array}{c} T_{s}=1536-\{415.3\left(\%C\right)+183.9\left(\%S\right)+12.3\left(\%Si\right)+6.8\left(\%Mn\right)\\ +4.3\left(\%Ni\right)+1.4\left(\%Cr\right)+30\left(\%P\right)\} \end{array}$	Empirical formula [24]
Thermal conductivity	$k_{s}=18.4+9.6\times 10^{-3}T$	Equivalent thermal conductivity [25]
	$k_{L}=mk_{s}$	
	$k_{s-L}=k_{s}-\frac{k_{s}-k_{L}}{T_{L}-T_{s}}\left(T-T_{s}\right)$	
Specific heat capacity ^1^	$C_{e}=\frac{C_{L}-C_{s}}{2}+\frac{L_{f}}{T_{L}-T_{s}}$	Equivalent specific heat capacity [26]
Latent heat of solidification	*L_f_* = 272 kJ·kg	Calculated [26]
Density	$\rho_{e}=\rho_{s}-\frac{\rho_{s}-\rho_{L}}{T_{L}-T_{s}}\left(T-T_{s}\right)$	Equivalent density [24]

^1^*C_L_* and *C_S_* are the specific melting points of the liquid and solid phases, respectively.

**Table 4:** Adjustment scheme of the secondary cooling water volume.

Cooling Section	Original Water Volume Inside (Outside) L·min^−1^	Weak Cold Water Inside (Outside) L·min^−1^	Strong Cold Water Inside (Outside) L·min^−1^
1N	128	77	145
1IO	150	120	200
2IO	187	90	350
3IO	212	119	420
4IO	202	114	359.5
5IO	172	128	230.9
6	66 (79)	56 (67.3)	78.1 (93.8)
7	102 (143)	99 (138.6)	105.1 (147.1)
8	65 (98)	68 (102.1)	61.7 (92.6)
9	52 (89)	52 (89)	52 (89)
10	60 (140)	50 (100)	50 (100)
11	60 (140)	70 (160)	20 (100)
Total water (L)	2145	1700	2694.8
Specific water	0.72 L·kg^−1^	0.56 L·kg^−1^	0.89 L·kg^−1^

**Table 5:** Industrial test results.

Defect	Test (One Strand)	Same Strand Comparison Test (One Strand)	Different Strand Comparison Test (Two Strands)
Corner transverse crack	0	2	10
Flawless	12	38	42
Total	12	40	52
Defect rate (%)	0	5	19
